# Estrogen-Receptor, Progesterone-Receptor and *HER2* Status Determination in Invasive Breast Cancer. Concordance between Immuno-Histochemistry and MapQuant™ Microarray Based Assay

**DOI:** 10.1371/journal.pone.0146474

**Published:** 2016-02-01

**Authors:** D. Mouttet, M. Laé, M. Caly, D. Gentien, S. Carpentier, H. Peyro-Saint-Paul, A. Vincent-Salomon, R. Rouzier, B. Sigal-Zafrani, X. Sastre-Garau, F. Reyal

**Affiliations:** 1 Department of Surgery, Institut Curie, Paris, France; 2 Residual Tumor and Response to Treatment Team, Institut Curie, Department of Translational Research, Paris, France; 3 Department of Tumor Biology, Institut Curie, Paris, France; 4 Department of Translational Research, Institut Curie, Paris, France; 5 QIAGEN Marseille SA, Marseille, France; University of Torino, ITALY

## Abstract

**Background:**

Hormone receptor status and *HER2* status are of critical interest in determining the prognosis of breast cancer patients. Their status is routinely assessed by immunohistochemistry (IHC). However, it is subject to intra-laboratory and inter-laboratory variability. The aim of our study was to compare the estrogen receptor, progesterone receptor and *HER2* status as determined by the MapQuant™ test to the routine immuno-histochemical tests in early stage invasive breast cancer in a large comprehensive cancer center.

**Patients and Methods:**

We retrospectively studied 163 invasive early-stage breast carcinoma with standard IHC status. The genomic status was determined using the MapQuant™ test providing the genomic grade index.

**Results:**

We found only 4 tumours out of 161 (2.5%) with discrepant IHC and genomic results concerning ER status. The concordance rate between the two methods was 97.5% and the Cohen’s Kappa coefficient was 0.89.

Comparison between the MapQuant™ PR status and the PR IHC status gave more discrepancies. The concordance rate between the two methods was 91.4% and the Cohen’s Kappa coefficient was 0.74.

The *HER2* MapQuant™ test was classified as « undetermined » in 2 out of 163 cases (1.2%). One *HER2* IHC-negative tumour was found positive with a high *HER2* MapQuant™ genomic score. The concordance rate between the two methods was 99.3% and the Cohen’s Kappa coefficient was 0.86.

**Conclusion:**

Our results show that the MapQuant™ assay, based on mRNA expression assay, provides an objective and quantitative assessment of Estrogen receptor, Progesterone receptor and *HER2* status in invasive breast cancer.

## Introduction

The Estrogen Receptor (ER) and Progesterone Receptor (PR) status are of critical interest in determining the prognosis of breast cancer patients and the potential benefit of adjuvant hormonal therapy. Their status is routinely assessed as well as the *HER2* status that is also a prognosis marker and determines patient’s eligibility to monoclonal antibody trastuzumab therapy.

The current standard methodology for measuring ER, PR and *HER2* status, is immunohistochemistry (IHC), with additional fluorescent *in situ* hybridization assay to clarify *HER2* immuno-histochemical status. It is subject to intra-laboratory and inter-laboratory variability. For instance, the inter-observer agreement in scoring hormone receptor status by IHC can vary from moderate to almost perfect (k = 0.78 to 0.85 for ER status, k = 0.71 to 0.72 for PR status [1] [2]). The discordance rate is mainly due to differences of interpretation of the specificity of staining and the histological structures after immunostaining. For example, Rhodes et al [3] found considerable inter-laboratory variation, especially for low estrogen receptor positivity, with a false negative rate between 30% and 60%. Arihiro et al [4] studied the inter-method variability due to effects of fixation, processing and different evaluation criteria (k = 0.34 for ER status, k = 0.45 for PR status). The larger study driven by Viale [5] comparing central versus local assessment of IHC hormone status (with a 10% cut-off for positivity), revealed a reclassification (after central reviewing) of 69.5% and 1.1% of the ER-negative and ER-positive tumours, and of 44.5% and 4.6% of PR-negative and PR-positive tumours. They concluded that central IHC should be performed whenever possible to correct the influence of the laboratory where the assay has been performed. The quality of *HER2* assays has been studied and a high degree of discordance between local and central laboratories has similarly been demonstrated (Table in S1 Table) [6–9].

Several studies investigated alternative methods to determine the hormonal receptor status (ER, PR) and *HER2* status with multi-genes signatures to address these limitations [10–14]. The genomic grade index (GGI) is a 97-gene measure of tumour grade. It is assessed by the MapQuant test, based on an Affymetrix microarray-based assay. Previous studies have shown that the genomic grade is an important tool to assess breast cancer tumour grade [15–17] and prognosis [18–21]. It has been demonstrated that the GGI could also predict response to chemotherapy [22, 23]. By using the MapQuant test, not only to determine the genomic grade but also to assess the prognostic and predictive markers *ER*, *PR* and *HER2*, we could potentially get a more reliable and informative determination of tumour characteristics compared to the immune-histochemistry assessments, therefore leading to a more reliable treatment decision.

The aim of our study was to compare the ER, PR and *HER2* status as determined by the MapQuant test to the routine immuno-histochemical tests in early stage invasive breast cancer in a large comprehensive cancer center.

## Patients and Methods

### Patients

The main inclusion criteria for the study were the absence of pathologic axillary lymph node involvement, a follow up above 10 years, and the absence of neoadjuvant therapy before surgery. Using these criteria, 456 early-stage (T1-T2 pN0) breast cancer patients treated between 1995 and 1996 could be retrieved from the Institut Curie database. From these cases, 169 flash-frozen samples stored at −80°C immediately after lumpectomy or mastectomy, and with more than 50% of tumor cells, were available. The histological features (histological type, histological grade assessed according to Elston and Ellis criteria, mitotic index, Ki67 proliferation index, ER status, PR status, *HER2* over expression status) were re-assessed for each sample by a large panel of pathologists experienced in breast pathology, using tissue sections (4 μm) prepared from a representative part of each tumour block fixed in AFA (Alcool/Formol/Acide acétique).

From the 169 cases available for analysis, 163 passed quality controls and constituted the reference cohort. The clinical and pathological features of these 163 cases are summarized in Table 1. Tumours corresponded mainly to ductal (78%) or lobular (13.5%) infiltrating carcinoma. All of them were free of axillary lymph node metastases. Tumours were classified as histological grade I in 32.5%, grade II in 43% and grade III in 24.5% of cases. Immuno-phenotyping showed that ER was expressed in 86% (140/163) of the tumors, PR in 68% (111/163), *HER2* in 6% (10/163) whereas 10% (17/163) remained negative for the three markers. The median follow-up duration was 154 months (6–182).

**Table 1:** Clinical and pathological features of 163 invasive early-stage breast carcinoma.

Clinical and Pathological Features. (N = 163)		
Clinical and histological features	Median (min-max)	Number of cases (%)
**Age at Diagnosis (years)**	53 (26–70)	
**Histological Subtype**		
**Infiltrating ductal**		127 (78%)
**Infiltrating lobular**		22 (13.5%)
**Mixed ductal/lobular**		6 (3.7%)
**Others**		8 (5%)
**Pathological Tumor Size (mm)**	20 (7–45)	
**Lympho Vascular invasion**		33 (20%)
**Histological grade**		
**Grade I**		53 (32.5%)
**Grade II**		70 (43%)
**Grade III**		40 (24.5%)
**Number of Mitoses (per ten HPF)**	6 (0–120)	
**Ki67 (percent)**	20 (0–100)	
**ER positive**		140 (86%)
**PR positive**		111 (68%)
**HER2 positive**		10 (6%)
**ER negative PR negative HER2 negative**		17 (10%)
**Hormone-therapy**		17 (10.4%)
**Chemotherapy**		11 (6.7%)
**Metastases Events**		29 (17.8%)
**Follow-up (months)**	154 (6–182)	

### Estrogen Receptor, Progesteron Receptor immunostaining

After rehydration and antigenic retrieval in citrate buffer (10 mM, pH 6.1), the tissue sections were stained for ER (ER, clone 6F11, Novocastra, 1/200), and PR (PR, clone 1A6, Novocastra, 1/200). Revelation of staining was performed using the Vectastain Elite ABC peroxidase mouse IgG kit (Vector Burlingame, CA) and diaminobenzidine (Dako A/S, Glostrup, Denmark) as chromogen. Positive and negative controls were included in each slide run. Cases were considered positive for ER and PR according to standardized guidelines using a cut-off of ≥10% stained tumour nuclei.

### *HER2* status

After rehydration and antigenic retrieval in citrate buffer (10 mM, pH 6.1), the tissue sections were stained for HER-2 (clone CB11, Novocastra, 1/1000). Revelation of staining was performed using the Vectastain Elite ABC peroxidase mouse IgG kit (Vector Burlingame, CA) and diaminobenzidine (Dako A/S, Glostrup, Denmark) as chromogen. Positive and negative controls were included in each slide run. The determination of *HER2* overexpression was determined according to GEFPICS (Groupe d’étude des facteurs pronostiques immunohistochimiques dans le cancer du sein, Unicancer) guidelines [24] with FISH performed in all cases of HER2 2+ result.

### MapQuant Dx protocol and Affymetrix data pre-processing

All 169 tumour samples available for genomic grade analysis contained more than 50% of cancer cells as assessed by H&E staining on frozen histological section of the samples used for the transcriptome analysis (manufacturer's recommendation: above 30%). RNA was extracted using Trizol method (Invitrogen) and purified using mirRNeasy kit (Qiagen). The concentration, integrity and purity of each RNA sample were measured using RNA 6000 LabChip kit with the Agilent 2100 Bioanalyser. The DNA microarrays used in this study were the Affymetrix HGU133 Plus 2.0 arrays (Affymetrix, Santa Clara, CA). Details of the RNA amplification, labeling and hybridization are available from the Affymetrix website (http://www.affymetrix.com). Chips were scanned using the GCS 3000 7G scanner (Affymetrix). Affymetrix quality controls variables were used to check data homogeneity. Profiles were normalized using RMAdx procedure (Robust Multi-array Average). RMA was applied to a reference set of microarrays (191 high-quality profiles), storing the parameters of the RMA fit. To process additional microarrays, these parameters are directly applied, without any re-estimation.

### ER, PR, *HER2* genomic status determination

MapQuant Dx Genomic Hormone Receptors (HR) quantifies the mRNA of 20 genes involved in breast-specific estrogen signaling and transcriptional cascades. The expression levels of these genes have been combined in an "ER score" and a "PR score" that best discriminate tumors expressing estrogen and/or progesterone receptors. Each score is based on a model fitted on 137 (76 ER- 0% vs 61 ER+ >60%) and 142 (93 PR- 0% vs 49 PR+ >30%) tumours respectively. The cut-off was set at 0, with score varying between -1.5 and +1.5. Based on this genomic score, ER and PR status are attributed to each tumour sample. A confidence interval (3:1 odds ratio of being ER- or ER+, PR- or PR+ respectively) was defined around the cut-off to ensure robustness and accuracy of status. For ER or PR scores into this confidence interval, the status is defined as “equivocal”.

MapQuant Dx genomic *HER2* quantifies the mRNA of 6 genes of the HER2 amplicon whose activity leads to HER2 protein expression at cell membrane level. The genomic *HER2* model was trained on 152 tumours (126 IHC 0 vs 26 IHC 3+). The cut-off was set at 0, with score varying between -3 and +3. Based on this genomic score, a *HER2* status is attributed to each tumour sample. A confidence interval (3:1 odds ratio of being *HER2*- or *HER2*+) was defined around the cut-off to ensure robustness and accuracy of status determination. For *HER2* scores into this confidence interval, the Her2 status is defined as “equivocal”.

### Statistical Analysis

Baseline characteristics were compared between groups using Chi-square or Fisher's exact tests for categorical variables and Student's t-tests for continuous variables. The analyses were performed using the R software (http://cran.r-project.org).

### Ethical approval

All experiments were performed retrospectively and in accordance with the French Bioethics Law 2004–800, the French National Institute of Cancer (INCa) Ethics Charter and after approval by the Institut Curie review board and ethics committee (Comit de Pilotage of the Groupe Sein). In the French legal context, our institutional review board waived the need for written informed consent from the participants. Moreover, women were informed of the research use of their tissues and did not declare any opposition for such researches. Data were analyzed anonymously.

## Results

We retrieved the equivocal MapQuant results from the cohort to determine the concordance rates.

### Comparison between MapQuant™ and IHC ER status

The ER Immunohistochemistry analysis showed that 86% of the tumours were classified as ER-positive (140/163). 142 out of 161 tumours were classified as genomic ER-positive (88%). The concordance rate between the two methods was 97.5% and the Cohen’s Kappa coefficient was 0.89.

The ER MapQuant test was classified as « equivocal » in 2 out of 163 cases (1%). Both tumours were IHC-positive with a 20% and 40% stained tumour nuclei respectively.

**Fig 1:**
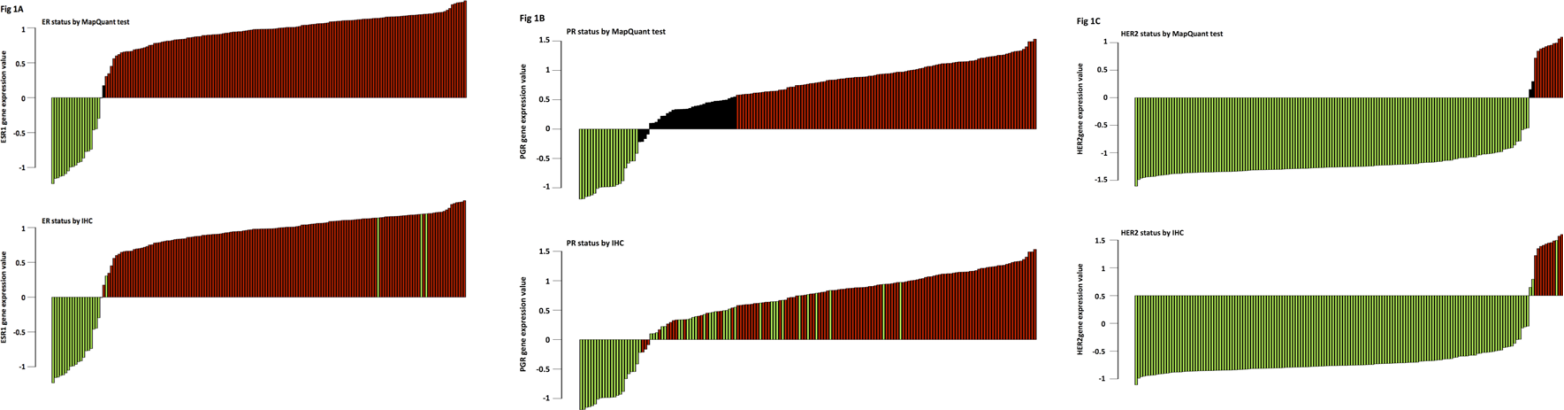
A: Estrogen-Receptor status (ER). Comparison between the Immunohistochemistry analysis (IHC) and the MapQuant test. Top. ER status determined by IHC referred to MapQuant test. Green: IHC-negative tumours. Red: IHC-positive tumours. Vertical axis: MapQuant test values. 4 IHC-negative cases were positive with the MapQuant test. Bottom. MapQuant determination of the ER status. Green: negative. Black: equivocal. Red: positive. Both equivocal cases corresponded to IHC-positive tumours. B: Progesterone-Receptor status (PR): comparison between the Immunohistochemistry analysis (IHC) and the MapQuant test. Top. PR status determined by IHC referred to MapQuant test. Green: IHC-negative tumours. Red: IHC-positive tumours. Vertical axis: MapQuant test values. 11 IHC-negative cases were positive with the MapQuant test. Bottom. Determination of the PR status by the MapQuant test. Green: negative. Black: equivocal. Red: positive. Equivocal cases corresponded to 15 IHC-positive tumours and 20 IHC-negative tumours. C: *HER2* status. Correlation between the Immunohistochemistry analysis (IHC) and the MapQuant test. Top. *HER2* status determined by IHC referred to MapQuant test. Green: IHC-negative tumours. Red: IHC-positive tumours. Vertical axis: MapQuant test values. Only one IHC-negative case was found positive with the MapQuant test. Bottom. Determination of the HER2 status by the MapQuant test. Green: negative. Black: equivocal. Red: positive. Equivocal cases corresponded to 2 IHC-negative tumours.

**Fig 2:**
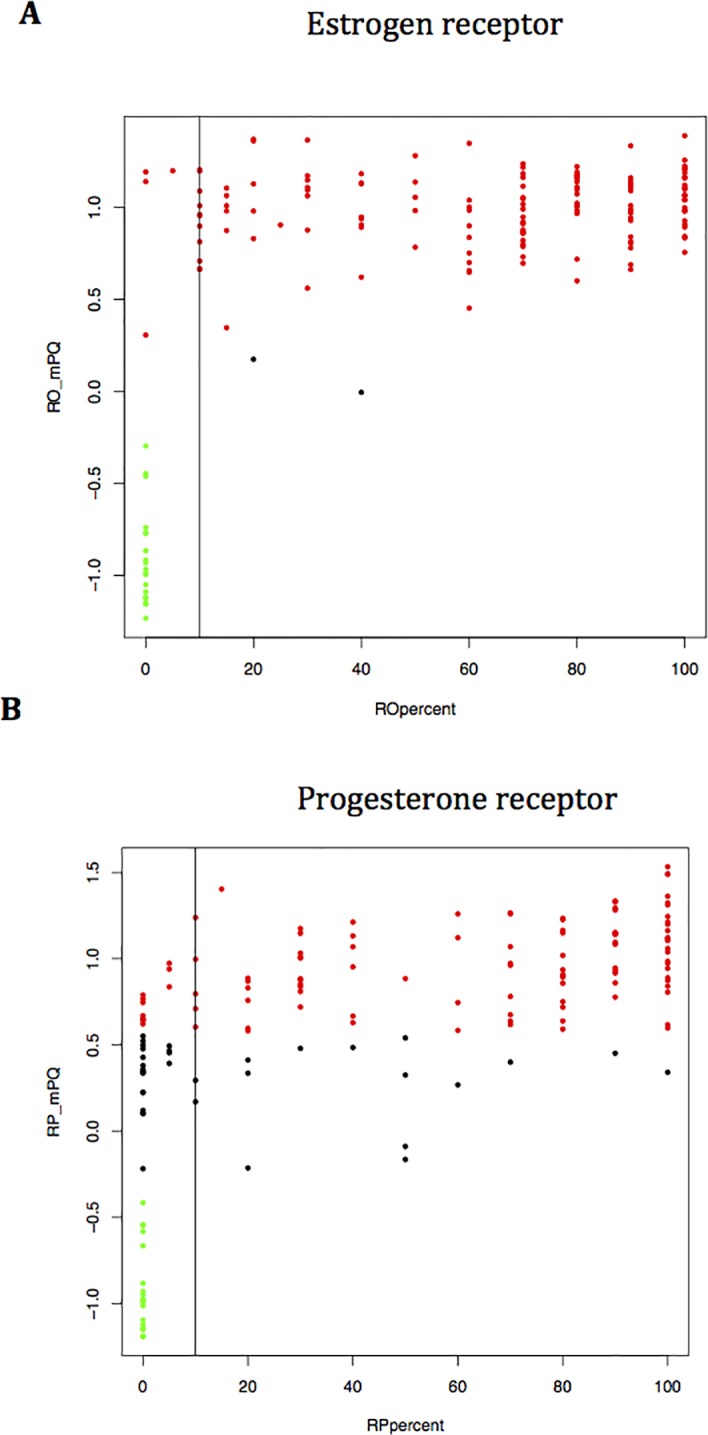
ER (A) and PR (B) expression by IHC and MapQuant test. Y axis: MapQuant values. X axis: Immunohistochemistry measure of ER and PR as percentage of stained tumour cells. Threshold for IHC positive sample is indicated by the vertical line (10%). MapQuant status. Green dot: negative. Black dot: equivocal. Red dot: positive.

**Fig 3:**
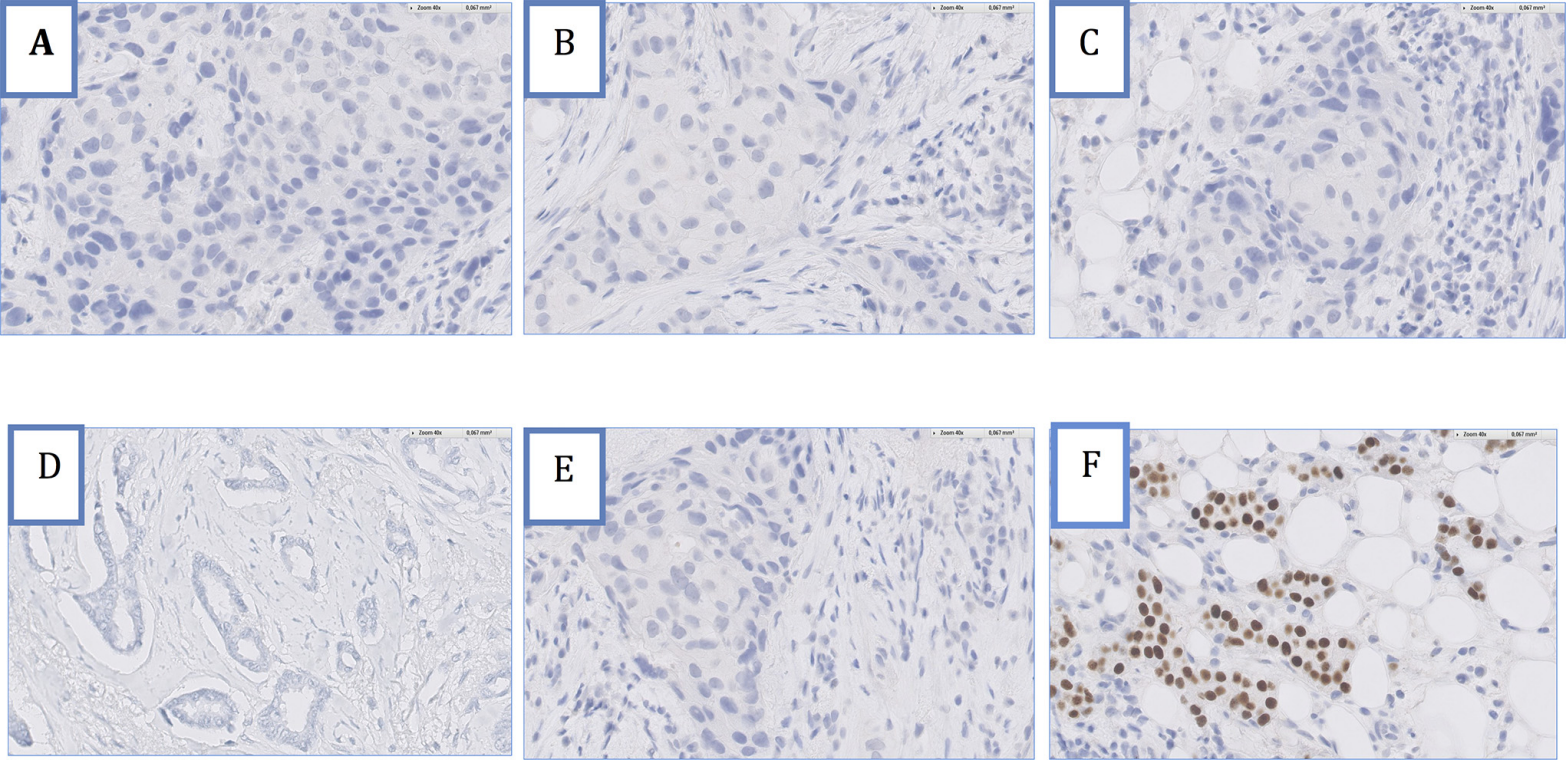
IHC pictures of 4 discordant cases for ER status. Patients (A-D) were IHC-negative/MapQuant-positive. Patient (E) was IHC-negative/MapQuant-negative. Patient (F) was IHC-positive 100%/MapQuant-positive.

We found only 4 tumours out of 161 (2.5%) with discrepant IHC and genomic results (Fig 1). ER MapQuant scores distribution related to the ER-IHC status is shown in Fig 2A. The four IHC-negative tumours with a positive ER MapQuant expression value showed an absence of stained tumour nuclei. Fig 3 shows the ER-IHC slides of these discordant cases compared with an ER-IHC-negative case also found negative with the ER MapQuant test. 3 out of these 4 ER-IHC negative discordant cases had a high ER MapQuant expression value above 1 (Fig 1).

### Comparison between the MapQuant™PR status and the PR IHC status

The PR Immunohistochemistry analysis showed that 68% of the tumours were PR-positive (111/163). 107 out of 128 tumours were classified as genomic PR-positive (83%). The concordance rate between the two methods was 91.4% and the Cohen’s Kappa coefficient was 0.74.

The PR MapQuant test was classified as « undetermined » in 35 out of 163 cases (21%). This group was equally composed of IHC-negative (57%) and IHC-positive (43%) tumours.

The PR status discrepancies were observed exclusively in the PR IHC-negative tumour subgroup. 11 out of 21 PR IHC-negative tumours (34%) were classified PR MapQuant positive. The PR MapQuant test value ranged between 0.5 and 1.0 (Figs 1 and 2B), while the percent positivity for IHC ranged from 10 to 100%. PR MapQuant expression values distribution related to the PR-IHC status is shown in Fig 2B.

### Comparison between the MapQuant™ *HER2* status and the *HER2* IHC status

The *HER2* Immunohistochemistry analysis showed that only 6% of the tumours were HER2-positive (10/163). 11 out of 161 tumours were classified as genomic HER2-positive (7%). The concordance rate between the two methods was 99.3% and the Cohen’s Kappa coefficient was 0.86.

The *HER2* MapQuant test was classified as « undetermined » in 2 out of 163 cases (1%). Both tumours were IHC-negative.

One *HER2* IHC-negative tumour was found positive with a high *HER2* MapQuant genomic score (Fig 1).

**Fig 4:**
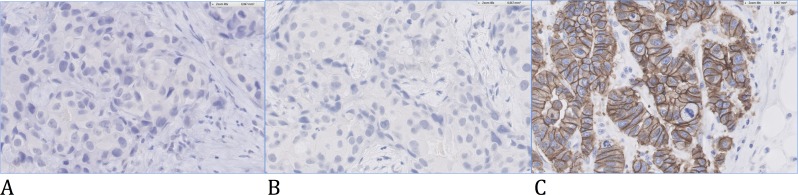
IHC picture of one discordant case for *HER2* status. Patient (A) was IHC-negative/MapQuant-negative. Patient (B) was IHC-negative/MapQuant-positive. Patient (C) was IHC-positive/MapQuant-positive.

Fig 4 shows the IHC slide of this discordant case for HER2 status.

### Treatment decision based on the IHC and genomic molecular subtype classification

ER, PR and *HER2* IHC status are surrogate markers able to identify the breast cancer molecular subtypes (Triple-negative, HER2pos ERpos, HER2pos ERneg, ERpos HER2neg). This classification is a major determinant of treatment decision. In our study, the IHC/genomic discordant results had almost no impact in terms of treatment choices. Only 2 out of 163 patients (1.2%) should have received a different treatment using the MapQuant results compared to the standard IHC tests. An IHC triple negative tumour (ER 5%, PR 5%) was re-classified as genomic ERpos/PRpos/HER2neg and would have received hormonal therapy. A second patient with an IHC ERpos/PRpos/HER2neg (HER2 IHC = 1+) tumour was re-classified as genomic ERpos/PRpos/HER2pos and would have received a targeted therapy (trastuzumab).

MapQuant test values of ER, PR and HER2 status compared to IHC are summarized in Table 2.

**Table 2:** MapQuant test values of Estrogen-Receptor (ER), Progesterone-Receptor (PR) and *HER2* status compared to immunohistochemistry (IHC).

		IHC status							
		ER		PR			HER2		
**MapQuant status**	**ER**	**+**	**-**	**PR**	**+**	**-**	**HER2**	**+**	**-**
	**+**	138	4	**+**	96	11	**+**	10	1
	**-**	0	19	**-**	0	21	**-**	0	150
	**ND**	2	0	**ND**	15	20	**ND**	0	2

ND = non determined or equivocal.

## Discussion

Our study was the first to determine the accuracy of the MapQuant assay to assess the ER, PR and *HER2* status.

Several studies investigated the accuracy of alternative methods for ER, PR and HER2 evaluations that may be more reliable and accurate than IHC in invasive breast cancers [10–14].

Currently, there are two commercially available prognostic breast cancer tests based on gene expression technology: 1) Oncotype DX (Genomic Health, Redwood City, California), 2) Mammaprint (Agendia BV, Amsterdam, the Netherlands) [25–31]. We compared the results of our study to published data (Table 3).

**Table 3:** Concordance (%) of the main Gene Expression Assays in determining ER, PR and *HER2* status compare to corresponding IHC reference.

Study	Test	Number of patients (N)	ER (95%CI)Kappa	PR (95%CI)Kappa	HER2 (95%CI)Kappa
**Our Study**	Mapquant	163	97%	91%	99%
			0.89	0.74	0.86
**Roepman et al**	Mammaprint	475	93%(91–95)	83%(80–86)	-
			0.79	0.65	
**Roepman et al**	Mammaprint	467	-	-	96%(94–98)
					0.88
**Gong et al**	Microarray	195	90% (85–94)	-	93% (89–96)
**Badve et al**	Oncotype DX	776	93% (91–95)	90% (88–92)	-
**Baehner et al**	Oncotype DX	568	-	-	97% (96–99)
**Dabbs et al**	Oncotype DX	843	-	-	98%

In our study, the genomic status correlated well with the IHC ER status. Our results are in agreement with Gong and colleagues [13], who investigated the use of Affymetrix microarrays for quantification of ESR1 and ERBB2 mRNA levels. In this paper, an ESR1mRNA cutoff value was identified which discriminates ER-positive tumours with an overall accuracy of 90% in the training set, 88% and 96% in two validation sets.

Roepman and colleagues [10] compared IHC with a second microarray-based mRNA expression level methodology (Mammaprint) and found a high level of concordance for ER status (93%). In their study, 4% of IHC-positive samples were classified negative using microarray, whereas in our study no IHC-positive samples were reclassified negative with the MapQuant test.

Viale et al [11] also found good concordance for ER status (98%) with the TargetPrint test in the first 800 patients enrolled in the MINDACT trial.

Badve and colleagues [11] compared a central 21-gene RT-PCR assay (OncotypeDX) to a local and a central IHC assay. They obtained good results for the ER status determination. Concordance between local IHC and central RT-PCR was 91%, and 93% between central IHC and central RT-PCR. Although concordance was high, IHC ER-negative cases that were RT-PCR positive (13% and 14% by local and central IHC) were more common than IHC-positive cases that were RT-PCR negative (1% and 5% by local and central IHC). Varga et al [11] detected a high concordance in hormone receptor and HER2 status between conventional IHC and OncotypeDX.

In our study, the PR status analysis showed the most discordant results between the two methodologies. 34% of the tumours classified PR negative by IHC were positive with the MapQuant test. Furthermore the « equivocal » group represented 21.4% of the tumours.

Our findings are in agreement with other studies on alternative gene expression technologies that report a lower concordance between PR mRNA levels and IHC. Badve and colleagues [11] found a concordance of 88% and 90% between local IHC, central IHC and central RT-PCR respectively (OncotypeDX). Roepman et al [10] found a concordance of 83% only between microarray (Mammaprint) and central IHC, similar with a lower concordance of 85% with the TargetPrint test.

Concerning the *HER2* status, there is a strong correlation between the two measures. We could see that using the genomic measure, we reclassified an IHC negative as genomic positive, which means that one extra patient should receive targeted therapy. The treatment decision for the equivocal group remains to be determined. Knowing the *HER2* oncogenic mechanism (gene amplification leading to increased mRNA expression and subsequently protein overexpression), one can understand the high concordance between the assessment of protein expression by IHC analysis and gene status by MapQuant test. Gong [13] also compared the determination of *HER2* status between IHC/FISH and Affymetrix gene expression profiling. They identified an overall accuracy of 93% in the training set, 89% and 90% in the two validation sets. The Mammaprint test also showed a 96% concordance for the *HER2* status determination [10]. Baehner et al [14] found an overall concordance of 97% and a positive agreement of 98% between *HER2* FISH assay and qRT-PCR using the Oncotype DX test.

Dabbs et al [12] studied the same test in a large independent multicenter study. They showed even with an overall concordance of more than 95%, that the percent positive agreement between the OncotypeDX test and IHC/FISH was less than 50% because of the small number of positive cases heavily diluted by the large number of negative patients in this biased population.

The MapQuant test is based on gene expression and provides information on mRNA expression, whereas IHC gives information on protein expression. As underlined by Allred in an editorial on problems and solutions in the evaluation of hormone receptors in breast cancers [32], there is no reason to expect similar results or performance from two different tests measuring either protein or mRNA expression, despite the fact that studies have found good concordance results especially for ER status between the two methods [10, 11].

The whole tumoural tissue (infiltrative carcinoma and DCIS (Ductal Carcinoma In Situ)) is extracted to obtain mRNA for the MapQuant test. So the ER, PR and HER2 status with MapQuant are made on the infiltrative carcinoma, DCIS and normal glands. Whereas the pathologist read only information about infiltrative carcinoma by doing IHC, excluding DCIS and normal breast tissue.

Plus, the MapQuant test is based on frozen tissue, whereas IHC is assessed on fixed tissues (FormalinFixedParaffinEmbedded). The two tests are based on two different tissue areas and the discordant results can be explained by the intratumoral heterogeneity.

The threshold for hormone receptors positivity in IHC can be set at 1 or 10% positive cells detection [13]. It is usually set at 10% in France. We re-analyzed the cases around/below the 10% cut-off to make our results more reliable for comparison with other studies (Table in S2 Table).

If we use a 1% cut-off to define a positive hormone receptor status:

- One case out of the 4 discordant cases would become ER positive (5% positivity) with IHC.
- 3 cases out of the 11 discordant cases would become PR positive (5% positivity) with IHC.

This new results doesn’t change significatively the concordance rates (3 instead of 4 discordant ER cases/ 8 instead of 11 discordant PR cases). The cut-off divergence doesn’t explane the high discrepancy in PR status between the 2 assays.

Concerning the lower PR concordance, Roepman et al [10] observed a higher proportion of cases that were IHC-positive/microarray-positive than IHC-positive/microarray-negative. They raised the possibility of a tumor subgroup that wouldn’t ‘express protein despite the presence of mRNA transcripts’.

In our study we observed more IHC-negative/MapQuant-positive and IHC-negative/MapQuant-undetermined results (6% and 12%) than IHC-positive/MapQuant-negative and IHC-positive/MapQuant-undetermined (0% and 9%).

Contrary to other studies, we didn’t find IHC-positive samples with MapQuant-negative result for each of the three biomarkers analysed (ER, PR, *HER2*). Few tumours were reclassified as MapQuant-undetermined, 1,4% (2/140) for ER status and 13,5% (15/111) for PR status, and none for the *HER2* status.

In our study, one patient would have been treated with trastuzumab therapy using the MapQuant-test. The major risk of this treatment is cardiotoxicity. However, the NSABP B-31 trial recently revealed that only 4.0% of patients who received trastuzumab in addition to adjuvant chemotherapy experienced a cardiac event after 7 years follow-up [33].

## Conclusion

In conclusion, our results show that the MapQuant assay, based on mRNA expression assay, provides an objective and quantitative assessment of Estrogen receptor, Progesterone receptor and *HER2* status in invasive breast cancer. The MapQuant test has similar performance compared to other gene expression profiling tests. It would need to be prospectively validated to prove its benefit and its medico economic impact beyond the use of standard clinico-pathological prognosis variables to guide the choice of adjuvant treatment.

## Supporting Information

S1 Table Concordance between local and central laboratories for HER2 assay. (TIFF)

S2 Table MapQuant test values of Estrogen-Receptor (ER), Progesterone-Receptor (PR) and HER2 status compared to immunohistochemistry (IHC) with a 1% cut-off positivity. (TIFF)
