# Supplementary figures and images for: Estrogen-Receptor, Progesterone-Receptor and HER2 Status Determination in Invasive Breast Cancer. Concordance between Immuno-Histochemistry and MapQuant™ Microarray Based Assay
Source: PLoS One. 2016 Feb 1;11(2):e0146474. doi: 10.1371/journal.pone.0146474 (PMC4735463; doi:10.1371/journal.pone.0146474)

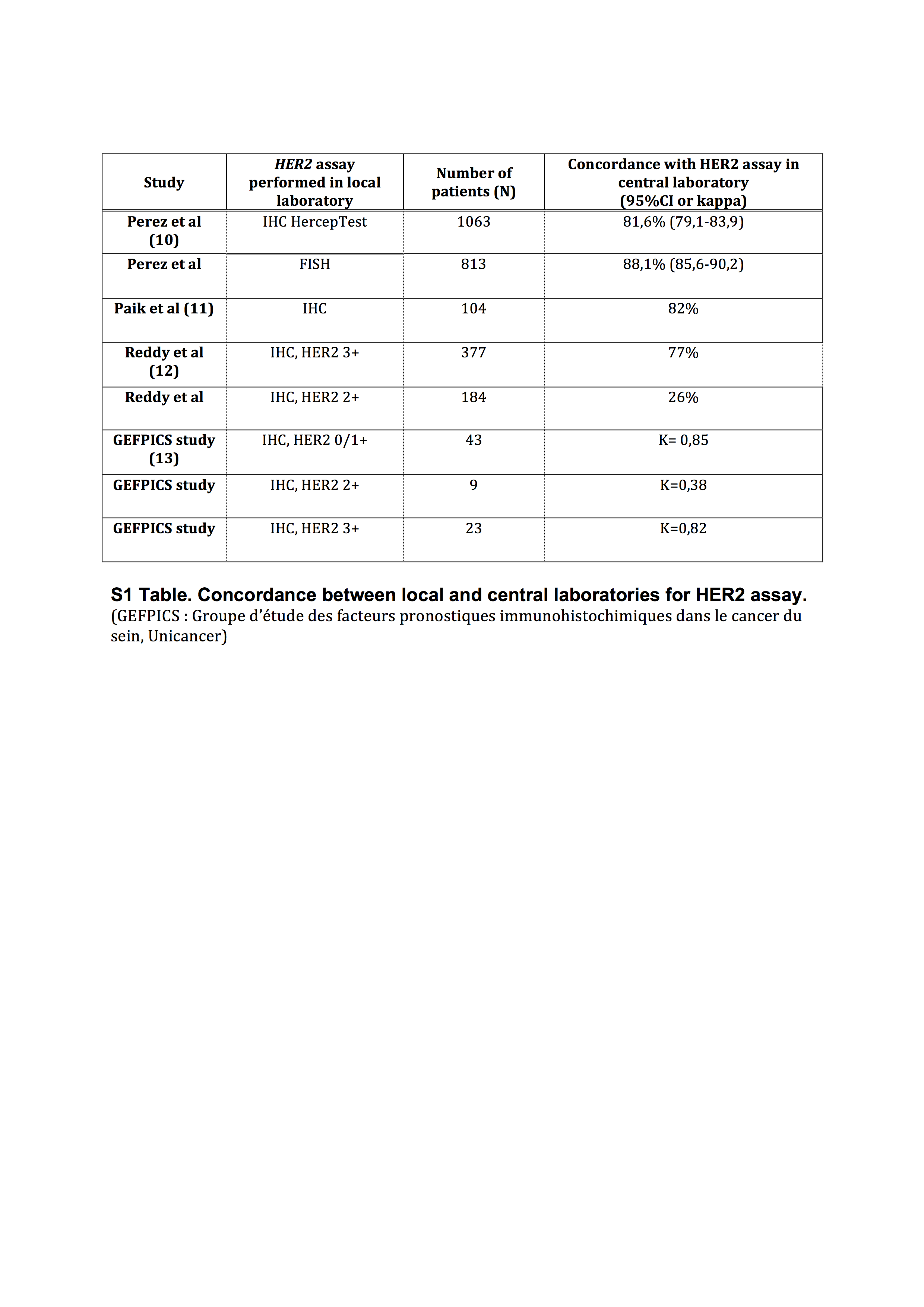

Supplement: S1 Table — (TIFF) [file pone.0146474.s001.tiff]

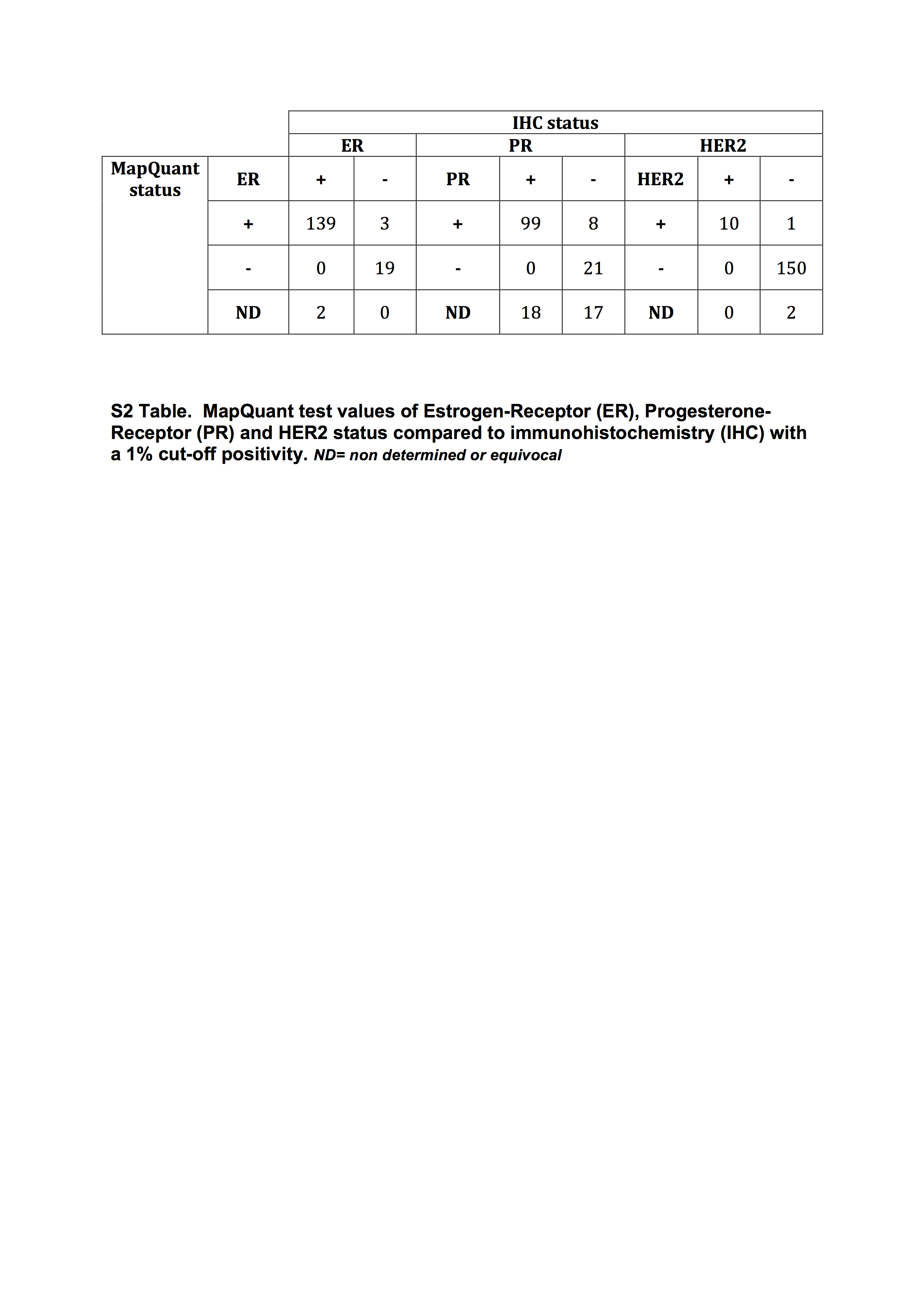

Supplement: S2 Table — (TIFF) [file pone.0146474.s002.tiff]
